# Supplementary material for: Complex‐centric proteome profiling by SEC‐SWATH‐MS
Source: Mol Syst Biol. 2019 Jan 14;15(1):e8438. doi: 10.15252/msb.20188438 (PMC6346213; doi:10.15252/msb.20188438)
Supplement: Supplementary file 8 — Dataset EV7 [file MSB-15-e8438-s008.zip › feature_plots_string/Q9GZL7.pdf]

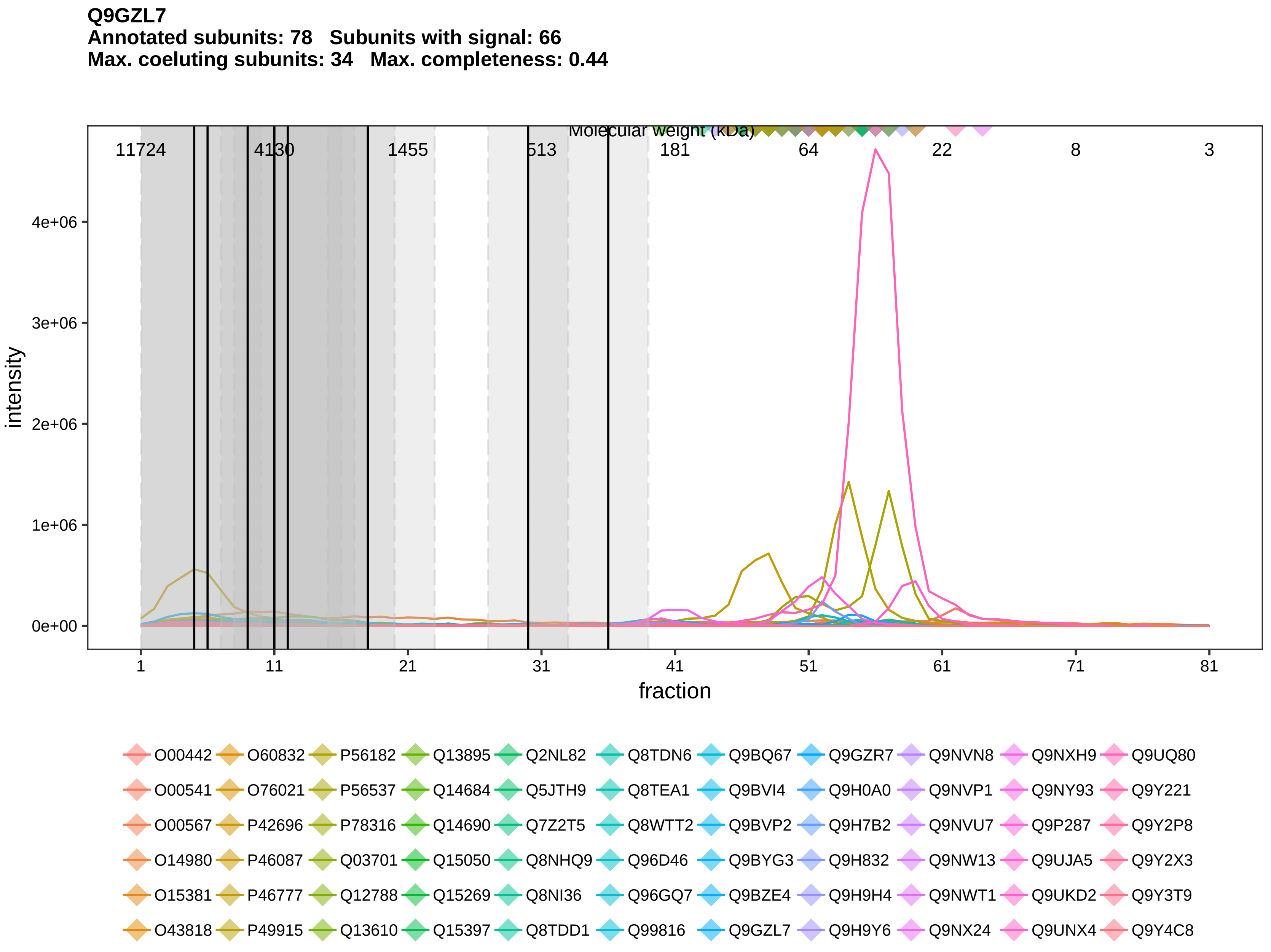
Q9GZL7
Annotated subunits: 78 Subunits with signal: 66
Max. coeluting subunits: 34 Max. completeness: 0.44
Molecular weight (kDa)
11724
4130
1455
513
181
64
22
8
3
intensity
4e+06
3e+06
2e+06
1e+06
0e+00
fraction
1
11
21
31
41
51
61
71
81
O00442
O60832
P56182
Q13895
Q2NL82
Q8TDN6
Q9BQ67
Q9GZR7
Q9NVN8
Q9NXH9
Q9UQ80
O00541
O76021
P56537
Q14684
Q5JTH9
Q8TEA1
Q9BVI4
Q9H0A0
Q9NVP1
Q9NY93
Q9Y221
O00567
P42696
P78316
Q14690
Q7Z2T5
Q8WTT2
Q9BVP2
Q9H7B2
Q9NVU7
Q9P287
Q9Y2P8
O14980
P46087
Q03701
Q15050
Q8NHQ9
Q96D46
Q9BYG3
Q9H832
Q9NW13
Q9UJA5
Q9Y2X3
O15381
P46777
Q12788
Q15269
Q8NI36
Q96GQ7
Q9BZE4
Q9H9H4
Q9NWT1
Q9UKD2
Q9Y3T9
O43818
P49915
Q13610
Q15397
Q8TDD1
Q99816
Q9GZL7
Q9H9Y6
Q9NX24
Q9UNX4
Q9Y4C8
